# Novel prognostic matrisome-related gene signature of head and neck squamous cell carcinoma

**DOI:** 10.3389/fcell.2022.884590

**Published:** 2022-08-23

**Authors:** Chao Huang, Yun Liang, Yi Dong, Li Huang, Anlei Li, Ran Du, Hao Huang

**Affiliations:** ^1^ Department of Nephrology, Xiangya Hospital, Central South University, Changsha, China; ^2^ Department of Otolaryngology-Head and Neck Surgery, Second Xiangya Hospital, Central South University, Changsha, China; ^3^ Department of Cell Biology, School of Life Sciences, Central South University, Changsha, China; ^4^ Hunan Key Laboratory of Organ Fibrosis, Central South University, Changsha, China; ^5^ National Clinical Research Center for Geriatric Disorders, Xiangya Hospital, Central South University, Changsha, China

**Keywords:** head and neck squamous cell carcinoma, differentially expressed genes, core matrisome gene, extracellular matrix, single-cell analysis, LAMB4

## Abstract

**Background:** Head and neck squamous cell carcinoma (HNSCC) is a common malignancy of the mucosal epithelium of the oral cavity, pharynx, and larynx. Laryngeal squamous cell carcinoma (LSCC) and oral squamous cell carcinoma are common HNSCC subtypes. Patients with metastatic HNSCC have a poor prognosis. Therefore, identifying molecular markers for the development and progression of HNSCC is essential for improving early diagnosis and predicting patient outcomes.

**Methods:** Gene expression RNA-Seq data and patient clinical traits were obtained from The Cancer Genome Atlas-Head and Neck Squamous Cell Carcinoma (TCGA-HNSC) and Gene Expression Omnibus databases. Differentially expressed gene (DEG) screening was performed using the TCGA-HNSC dataset. Intersection analysis between the DEGs and a list of core matrisome genes obtained from the Matrisome Project was used to identify differentially expressed matrisome genes. A prognostic model was established using univariate and multivariate Cox regression analyses, least absolute shrinkage, and selection operator (LASSO) regression analysis. Immune landscape analysis was performed based on the single-sample gene set enrichment analysis algorithm, Gene Ontology, Kyoto Encyclopedia of Genes and Genomes, prognostic value, receiver operating characteristic curve analysis, and gene mutation analyses. Immunohistochemical results regarding prognostic protein levels were obtained from the Human Protein Atlas. Single-gene RNA-sequencing data were obtained from GSE150321 and GSE172577 datasets. CCK-8 and Transwell assays were used to confirm cell proliferation and migration.

**Results:** A total of 1,779 DEGs, including 939 upregulated and 840 downregulated genes, between tumor and normal samples were identified using the TCGA-HNSC microarray data. Intersection analysis revealed 52 differentially expressed matrisome-related genes. After performing univariate and multivariate Cox regression and LASSO analyses, a novel prognostic model based on six matrisome genes (*FN1*, *LAMB4*, *LAMB3*, *DMP1*, *CHAD*, and *MMRN1*) for HNSCC was established. This risk model can successfully predict HNSCC survival. The high-risk group had worse prognoses and higher enrichment of pathways related to cancer development than the low-risk group. Silencing *LAMB4* in HNSCC cell lines promoted cell proliferation and migration.

**Conclusion:** This study provides a novel prognostic model for HNSCC. Thus, *FN1*, *LAMB4*, *LAMB3*, *DMP1*, *CHAD*, and *MMRN1* may be the promising biomarkers for clinical practice.

## 1 Introduction

Head and neck squamous cell carcinoma (HNSCC) is a common malignancy derived from the mucosal epithelium of the oral cavity, pharynx, and larynx. HNSCC has a yearly global incidence of over 830,000 cases and over 430,000 deaths (Bray et al., 2018; Cramer et al., 2019). Approximately 75% of HNSCC incidence is associated with tobacco-derived carcinogens or excessive alcohol consumption (Johnson et al., 2020). The most common HNSCC subtypes are laryngeal squamous cell carcinoma (LSCC), oral squamous cell carcinoma (OSCC), and nasopharyngeal carcinoma (Shield et al., 2017; Solomon et al., 2018).

LSCC accounts for approximately 20% of all head and neck malignancies and is the second most common malignancy after lung cancer among upper aerodigestive tract tumors (Karatzanis et al., 2014). The symptoms and prognosis of LSCC vary depending on its site of origin. Overall, the outcomes of supraglottic and subglottic LSCC are generally poor and significantly worse than those of glottic laryngeal cancer (Coskun et al., 2019). Despite advances in the diagnosis and treatment of LSCC, the 5-years overall survival rate is approximately 50%, which is a modest improvement compared to the survival rate of patients diagnosed with other tumors (Koontongkaew, 2013). Therefore, identifying molecular markers involved in the development and progression of LSCC is essential to enhance the understanding of the mechanisms underlying these processes and improve early diagnosis and prognosis (Yang et al., 2020).

OSCC incidence continues to rise, affecting more than 300,000 people annually. It is the sixth largest and one of the most prevalent types of cancer in the world (Zhao et al., 2021; Sherin et al., 2008). As OSCC is highly aggressive, patients are usually presented with disease progression during diagnosis and treatment. The patients have a recurrence rate of over 50% (Kapoor et al., 2012). It has been confirmed that the poor prognosis of OSCC is associated with the neck lymph node metastasis (Dhumal et al., 2022; Montero and Patel, 2015), of which 68% develops in the lungs (Irani, 2016). Therefore, identifying common molecular markers in LSCC and OSCC can not only discover the relationship between the two diseases, but also play a vital role in their diagnosis and treatment.

The extracellular matrix (ECM) is a complex network composed of biological macromolecules synthesized intracellularly by cells and secreted extracellularly to reside on the cell surface or between cells (Theocharis et al., 2019). The core matrisome comprises over 250 unique matrix genes that are classified into collagens, glycoproteins, and proteoglycans (Naba et al., 2016). The expression profiles of ECM proteins in tumors are significantly different from those in the normal tissues (Marozzi et al., 2021). ECM remodeling in cancer is a critical component of the tumor microenvironment (TME) and is also an important driving force for the development of malignant tumors. ECM deposition, remodeling, and cross-linking are closely related to the development and prognosis of tumors (Dongre and Weinberg, 2019; Winkler et al., 2020). However, research on the role of the matrisome in HNSCC is requires further investigation.

This study aimed to develop a prognostic model based on matrisome genes to predict patient survival using The Cancer Genome Atlas-Head and Neck Squamous Cell Carcinoma (TCGA-HNSC) dataset. The results were validated using four independent datasets of LSCC or OSCC, including integrated single-cell RNA-sequencing data (scRNA-Seq) from the Gene Expression Omnibus (GEO) database. The present study aimed to reveal the relationship between ECM remodeling and HNSCC pathogenesis as well as to provide insights into novel therapeutic targets for the treatment of HNSCC.

## 2 Methods

### 2.1 Data collection and single-cell RNA-sequencing data analysis

**FIGURE 1:**
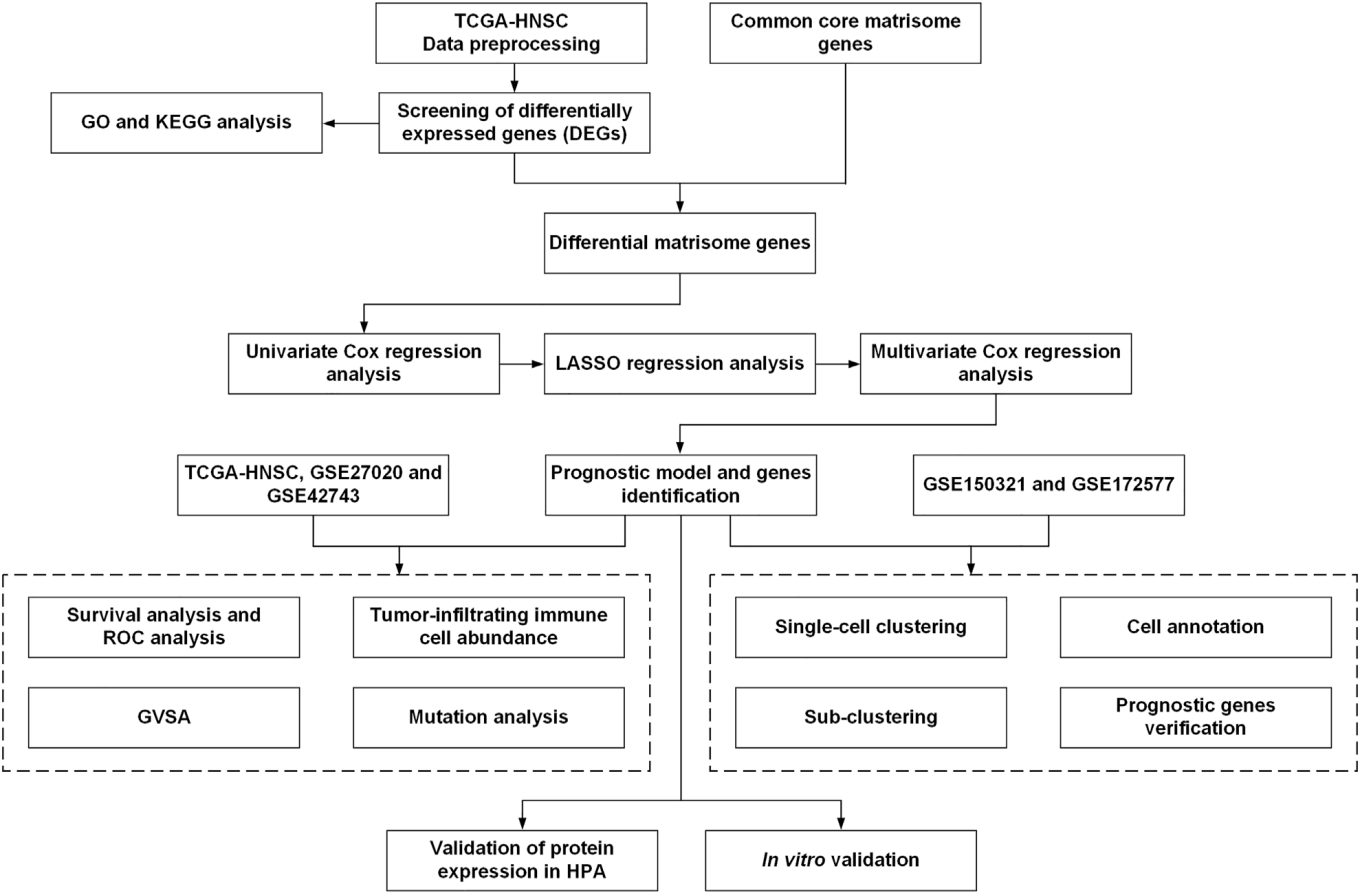
Schematic diagram of the workflow of the present study.

The workflow of this study is illustrated in Figure 1. Gene expression RNA-Seq data, including count and TPM data, were obtained from the University of California, Santa Cruz (UCSC) browser Xena (http://xena.ucsc.edu/) and were associated with TCGA-HNSC clinical information. Gene expression microarray datasets (log_2_-transformed Robust Multi-Array normalized data) and their associated prognostic datasets, GSE27020 (Fountzilas et al., 2013) and GSE42743 (Lohavanichbutr et al., 2013), were selected and downloaded from the GEO database (https://www.ncbi.nlm.nih.gov/geo/). These GEO datasets were annotated with gene symbols using information from the GPL96 Affymetrix Human Genome U133 A Array and GPL570 Affymetrix Human Genome U133 Plus 2.0 Array platform files, respectively. All the data were processed using R (version 4.0.4) and RStudio (version 1.2.5033). TCGA-HNSC contained 502 HNSCC tumor 44 normal tissues, GSE27020 contained 109 LSCC samples with survival data, and GSE42743 contained 74 OSCC and 29 normal samples with survival data (Supplementary Table S1).

ScRNA-Seq data from the GSE150321 (Song et al., 2020) and GSE172577 (Peng et al., 2021) datasets were obtained from the GEO database. The R packages “Seurat” (version 4.0.2) and “scCancer” (Guo et al., 2021) were used to process the data. The uniform manifold approximation and projection (UMAP) algorithm was used to explore and visualize cluster classifications across the cell samples.

A list of 274 core matrisome genes was obtained from the Matrisome Project (http://matrisomeproject.mit.edu) (Shao et al., 2020). Genes with expression in TCGA-HNSC, GSE27020, and GSE42743 datasets were included for further analysis. A total of 190 common core matrisome genes (Supplementary Table S2), including 33 collagens, 127 ECM glycoproteins, and 30 proteoglycan-coding genes, were selected.

### 2.2 Identification of differentially expressed genes

Differential expression analysis was performed using edgeR (Robinson et al., 2010) in the OmicShare tool, a free online platform for data analysis (www.omicshare.com/tools). Protein-coding genes with mean counts of >5 were selected. The default parameters of edgeR were used, and DEGs were selected according to log_2_ |fold–change (FC)| ≥ 2.0 and Q value >0.05. As there were no replicates in this study, the biological coefficient of variation (BCV), which is the square root of dispersion, was set to 0.01 following the suggestion from the edgeR official manual.

### 2.3 Pathway and process enrichment analysis

The Metascape tool (Zhou et al., 2019b) (https://metascape.org) was used to perform Gene Ontology (GO) analysis for biological processes (BP), along with Kyoto Encyclopedia of Genes and Genomes (KEGG) pathway enrichment analysis. Statistical significance was set at *p* < 0.01.

### 2.4 Least absolute shrinkage and selection operator and univariate and multivariate cox regression analyses

Univariate Cox regression analysis was performed to screen the differential matrisome genes that were significantly associated with overall survival (OS) in the TCGA-HNSC dataset. Genes with *p* < 0.1 were included for subsequent research.

LASSO and multivariate Cox regression model was widely used for high-dimensional predictor identification. LASSO regression analysis was performed using the “glmnet” R package (version 4.1-2). For multivariate Cox regression model analysis, OS-associated matrisome genes were used to select the significant genes associated with the OS of patients with HNSCC according to the coefficient value. These factors were incorporated in the multivariate Cox regression model to construct the HNSCC prognostic signature. The risk score was calculated using the following formula:

$$\text{Risk Score}=\overset{n}{\underset{i=1}{\sum }}\beta_{i}\times Exp_{i}$$

In this formula, *β* denotes the regression coefficient, and *Exp* denotes the expression levels of each core matrisome gene, *i* (Hou et al., 2020; Huang et al., 2021b). Samples in the TCGA-HNSC cohort were divided into high- or low-risk groups depending on their median risk scores. Receiver operating characteristic (ROC) and Kaplan–Meier analyses were conducted between the high- and low-risk groups. The R packages “pROC” (version 1.18.0), “timeROC” (version 0.4), “survival” (version 3.2-13) and “survminer” (version 0.4.9) were used for visualization.

### 2.5 Evaluation of tumor-infiltrating immune cells

Single-sample gene set enrichment analysis (ssGSEA) was used to evaluate the relative proportion of 23 infiltrating immune cell types in TCGA-HNSC, including adaptive immune cells (activated B, activated CD4^+^ T, activated CD8^+^ T, gamma delta T, immature B, regulatory T, T follicular helper, type 1 T helper [Th1], Th17, and Th2 cells), and innate immune cells (activated dendritic cells [DCs], CD56bright natural killer cells, CD56dim natural killer cells, eosinophils, immature DCs, macrophages, mast cells, myeloid-derived suppressor cells (MDSCs), monocytes, natural killer cells, natural killer T cells, neutrophils, and plasmacytoid DCs). A bar plot was drawn to evaluate the differences in the composition of these 23 types of tumor-infiltrating immune cells between the high and low-risk groups. The correlation analysis of the relationship between risk score and immune cells was visualized by the “corrplot” R package (version 0.92).

### 2.6 Mutation analysis

The R package “maftools” (version 2.6.05) was used to calculate the tumor mutation burden score for each sample from the high- and the low-risk groups of the TCGA-HNSC dataset and generate the oncoplot waterfall plot.

### 2.7 Functional enrichment analysis of the prognostic genes using gene set variation analysis

KEGG analysis was conducted on the high- and low-risk groups using GSVA. Reference information was downloaded from the Molecular Signature Database v7.4 (MSigDB v7.4, http://software.broadinstitute.org/gsea/msigdb/index.jsp). Statistical significance was set at adjusted-*p* < 0.05.

### 2.8 Immunohistochemistry analysis and protein expression level verification using the human protein atlas database

The HPA is a database that maps all human proteins in cells, tissues, and organs using an integration of various omics technologies (https://www.proteinatlas.org/). We verified the protein expression levels of survival-related matrisome genes by IHC using the HPA database.

### 2.9 Cell culture and transfection

The laryngeal cancer cell line AMC-HN-8 and OSCC cell line JHU011 were acquired from the Department of Otolaryngology-Head and Neck Surgery, Xiangya Hospital, Central South University. The cell lines were maintained in Dulbecco’s Modified Eagle’s Medium (DMEM) with high glucose (Procell Life Science&Technology Co., Ltd., Wuhan, China) and 10% fetal bovine serum (FBS) (Procell Life Science&Technology Co., Ltd.). Cells were maintained at 37°C in a humidified incubator with 5% CO_2_.

The *LAMB4* siRNA was produced by GenePharma Inc. (Suzhou, China). The *LAMB4* siRNA sequence was 5′-GCC​UUC​GAU​GUU​UGC​ACA​ATT-3′, and the control siRNA sequence was 5′-UUC​UCC​GAA​CGU​GUC​ACG​UTT-3′. The siRNA was transfected into AMC-HN-8 or JHU011 using Lipofectamine 3,000 Reagent (Invitrogen, United States) with the Opti-MEM medium (Gibco, Waltham, MA, United States).

### 2.10 RNA isolation and RT-PCR

The cell sample total RNA was extracted using the TRIzol reagent (Solarbio, Beijing, China) and subjected to reverse transcription with random primers using the RevertAid First Strand cDNA Synthesis Kit (Thermo Fisher Scientific, United States). The expression level of the targeted genes was measured with the Maxima SYBR Green/ROX qPCR Mix (Thermo Fisher Scientific) using a real-time PCR system (Roche, Basel, Switzerland). The relative RNA expression levels were calculated using the 2^(−△△CT)^ method. The 18 s rRNA was used as an internal control. The primer sequences of *LAMB4* were 5′-AAA​GAG​AAC​GTG​GAA​GGA​GC-3′ (forward) and 5′-TCA​CAG​GTC​AAG​AAT​GGC​AG-3′ (reverse), and the primer sequences of 18 s rRNA were 5′-CCT​GGA​TAC​CGC​AGC​TAG​GA-3′ (forward) and 5′-GCG​GCG​CAA​TAC​GAA​TGC​C-3′ (reverse).

### 2.11 CCK-8 assay

A total of 2,000 cells per well were seeded into 96-well plates. For each well, 10 μL CCK-8 reagent (Solarbio, Beijing, China) was added into the medium. After incubation at 37°C for 2 h, the optical density (OD) was measured at different time points at 450 nm.

### 2.12 Transwell assay

A total of 2.5 × 10^5^ cells per well were suspended in DMEM and seeded into a 24-well 8.0-mm transwell top chamber (Jet Biofil, Guangzhou, China). DMEM supplemented with 12% FBS was added to the bottom chambers. After incubation at 37°C for 16 h, the cells at the top chambers were fixed with 4% paraformaldehyde for 30 min, followed by permeabilization with methyl alcohol for 20 min. The cells were then stained with 0.1% crystal violet (Solarbio, Beijing, China) for 15 min. Cells that did not migrate through the pores were removed using a cotton swab. Cells on the bottom of the chamber were counted using an inverted phase-contrast microscope at low magnifications ( × 10) (at least three randomly selected fields were quantified).

### 2.13 Statistical analysis

The statistical analyses were performed using R software (version 4.0.4) or GraphPad Prism (version 8.0). Gene expression levels were compared using Student’s *t*-test. *P* < 0.05 was considered statistically significant. *p < 0.05; **p < 0.01; ***p < 0.001.

## 3 Results

### 3.1 Differentially expressed genes screening

**FIGURE 2:**
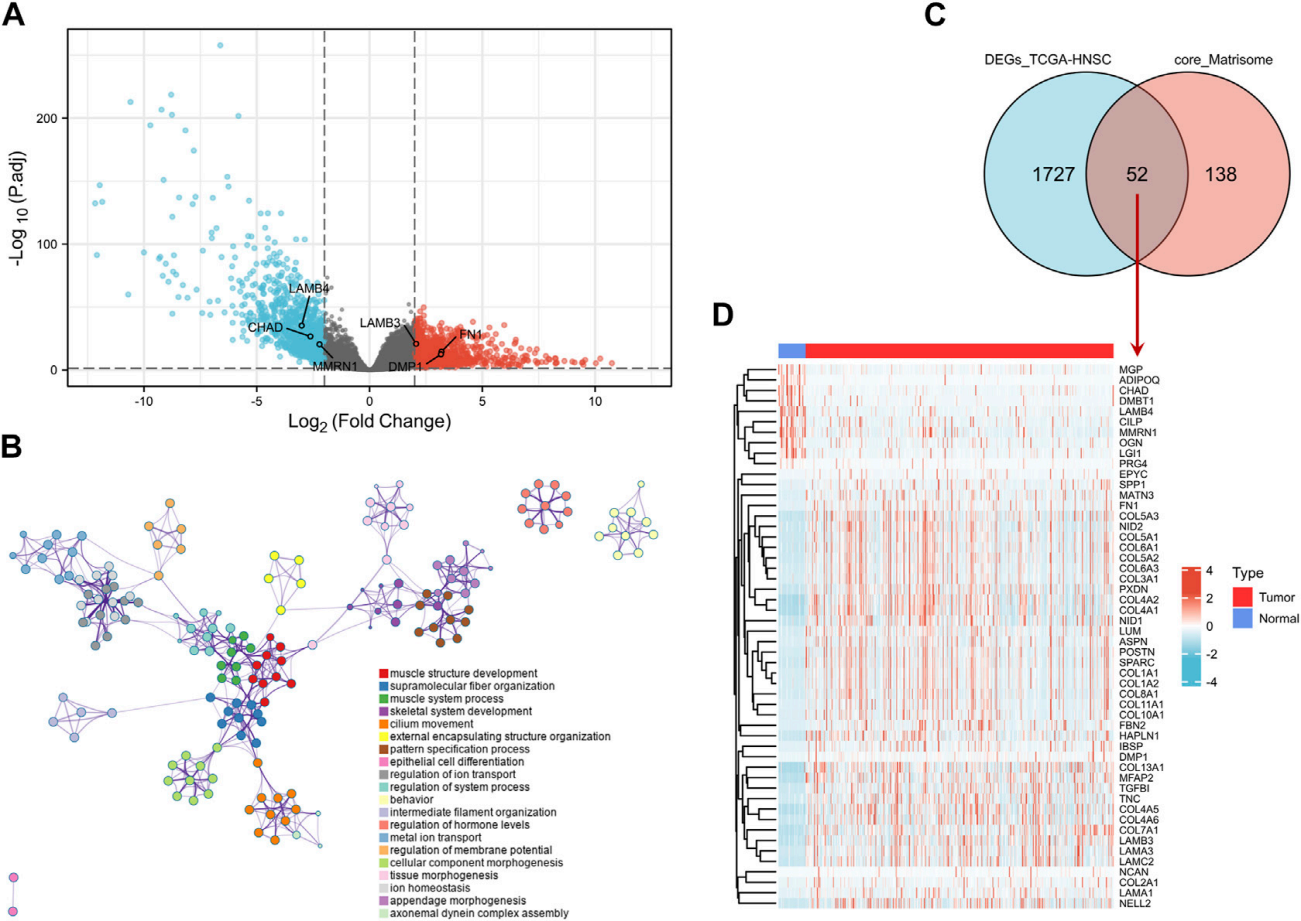
Screening of DEGs. **(A)** Volcano map of DEGs between HNSCC and normal samples in the TCGA-HNSC dataset. The red plots in the volcano represent upregulated genes and the blue points represent downregulated genes. **(B)** GO BP analysis of the DEGs. **(C)** Venn plot of the DEGs in TCGA-HNSC dataset and common core matrisome genes. **(D)** Heatmap of 52 differentially expressed matrisome-related genes in TCGA-HNSC. DEG, differentially expressed gene; BP, biological process; GO, Gene Ontology; HNSCC, head and neck squamous cell carcinoma.

DEG analysis was conducted by genetic screening of TCGA-HNSC microarray data with a threshold of |log_2_(FC)| > 2.0 and an adjusted-*p* < 0.05. A total of 1,779 DEGs between tumor and normal samples were identified, including 939 upregulated genes and 840 downregulated genes (Figure 2A).

The DEGs were mostly enriched in “muscle structure development,” “supramolecular fiber organization,” “skeletal system development,” “cilium movement,” “pattern specification process,” and “extracellular matrix organization” in GO BP analysis (Figure 2B and Supplementary Figure S1). In the KEGG analysis, DEGs were enriched in “protein digestion and absorption,” “salivary secretion,” “ECM-receptor interaction,” “calcium signaling pathway,” “focal adhesion,” and “hypertrophic cardiomyopathy” (Supplementary Figures S2A,B).

Intersection analysis of the DEGs in TCGA-HNSC and 190 common core matrisome genes identified 52 differentially expressed matrisome-related genes (Figures 2C,D). These genes were used for subsequent analysis.

### 3.2 Establishment of a prognostic model based on differentially expressed matrisome genes

To establish a prognostic assessment model for patients with HNSCC, we selected the TCGA-HNSC dataset (only tumor samples with survival data, *N* = 502) as the training cohort and GSE27020 (LSCC samples, *N* = 109) and GSE42743 (OSCC samples, *N* = 74) datasets as testing cohorts. Univariate Cox regression analysis of the 52 differentially expressed matrisome genes was performed to evaluate the OS of the samples from the training cohort (Supplementary Table S3). Twelve genes (*MATN3*, *LAMC2*, *FN1*, *SPP1*, *LAMB4*, *LAMB3*, *DMP1*, *IBSP*, *DMBT1*, *TGFBI*, *CHAD*, and *MMRN1*) with *p* < 0.1 were then included for LASSO analysis. All 12 matrisome genes achieved minimum partial expression (Supplementary Figure S2A,B) and were further incorporated into multivariate Cox regression analysis with their expression levels and prognostic data to identify genes involved in signature construction. Multivariate Cox regression analysis established a prognostic model consisting of a risk signature comprising six genes (*FN1*, *LAMB4*, *LAMB3*, *DMP1*, *CHAD*, and *MMRN1*). The formula for the risk score calculation was as follows: risk score = 0.075123 × *FN1* + (−0.348,219) × *LAMB4* + 0.090359 × *LAMB3* + 0.363,187 × *DMP1* + (−0.314,325) × *CHAD* + (−0.125,206) × *MMRN1* (Supplementary Figure S3).

**FIGURE 3:**
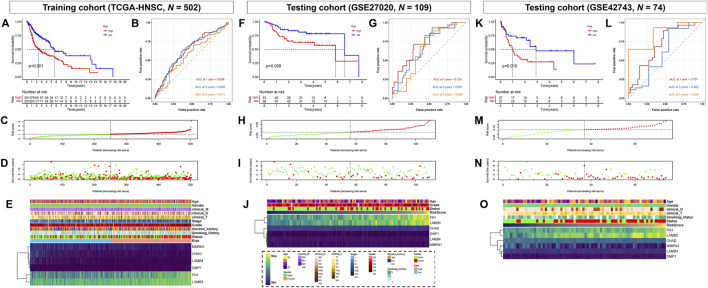
Prognostic analysis of the key genes. **(A)** Overall survival between high- and low-risk score groups in the training cohort (TCGA-HNSC, *N* = 502). **(B)** ROC curve analysis between high- and low-risk score groups in the training cohort. **(C)** Risk score distribution of patients in the training cohort. Green dots represent individuals in the low-risk group, and red dots represent those in the high-risk group. **(D)** Scatter plots of survival status of patients in the training cohort. Green dots represent survival individuals and red represent death individuals. **(E)** Expression patterns of risk genes in the training cohort. **(F)** Overall survival between high- and low-risk score groups in the testing cohort (GSE27020, *N* = 109). **(G)** ROC curve analysis between high- and low-risk score groups in the testing cohort (GSE27020). **(H)** Risk score distribution of patients in the testing cohort (GSE27020). Green dots represent individuals in the low-risk group and red represent in the high-risk group. **(I)** Scatter plots of survival status of patients in the testing cohort (GSE27020). Green dots represent survival individuals and red represent death individuals. **(J)** Expression patterns of risk genes in the testing cohort (GSE27020). **(K)** Overall survival between high- and low-risk score groups in the testing cohort (GSE42743, *N* = 103). **(L)** ROC curve analysis between high- and low-risk score groups in the testing cohort (GSE42743). **(M)** Risk score distribution of patients in the testing cohort (GSE42743). Green dots represent individuals in the low-risk group and red represent in the high-risk group. **(N)** Scatter plots of survival status of patients in the testing cohort (GSE42743). Green dots represent survival individuals and red represents death individuals. **(O)** Expression patterns of risk genes in the testing cohort (GSE42743). DEG, differentially expressed gene; ROC, receiver operating characteristic; AUC, area under the curve.

According to the risk score, samples from the training and testing cohorts were divided into two groups, a high-risk and low-risk group, based on a cutoff value of 50% (Figures 3A−O). Kaplan–Meier analysis showed that patients with high-risk scores had significantly shorter survival times than those in the low-risk group, both in the training (TCGA-HNSC) and testing cohorts (GSE27020 and GSE42743) (Figures 3A,F,K). In addition, the area under the curve (AUC) values of the ROC curves for the 1-, 2-, and 5-years survival rates were 0.636, 0.635, and 0.571, respectively, indicating that the risk score can be used to predict prognosis in the training cohort (TCGA-HNSC) (Figure 3B). In the testing cohorts, the AUC values for the 1-, 2-, and 5-years survival rates were 0.724, 0.691, and 0.650 in GSE27020 (Figure 3G) and 0.707, 0.602 and 0.859 in GSE42743 (Figure 3L), respectively. Additionally, all six genes (*FN1*, *LAMB4*, *LAMB3*, *DMP1*, *CHAD*, and *MMRN1*) were significantly associated with poor prognosis and unhealthy living habits in both the training (TCGA-HNSC) and the testing cohorts (GSE27020 and GSE42743) (Figures 3E,J,O). Therefore, a matrisome-associated prognostic model for HNSCC was established and verified.

### 3.3 Comparison of tumor-infiltrating immune cell landscapes between high- and low- risk groups

**FIGURE 4:**
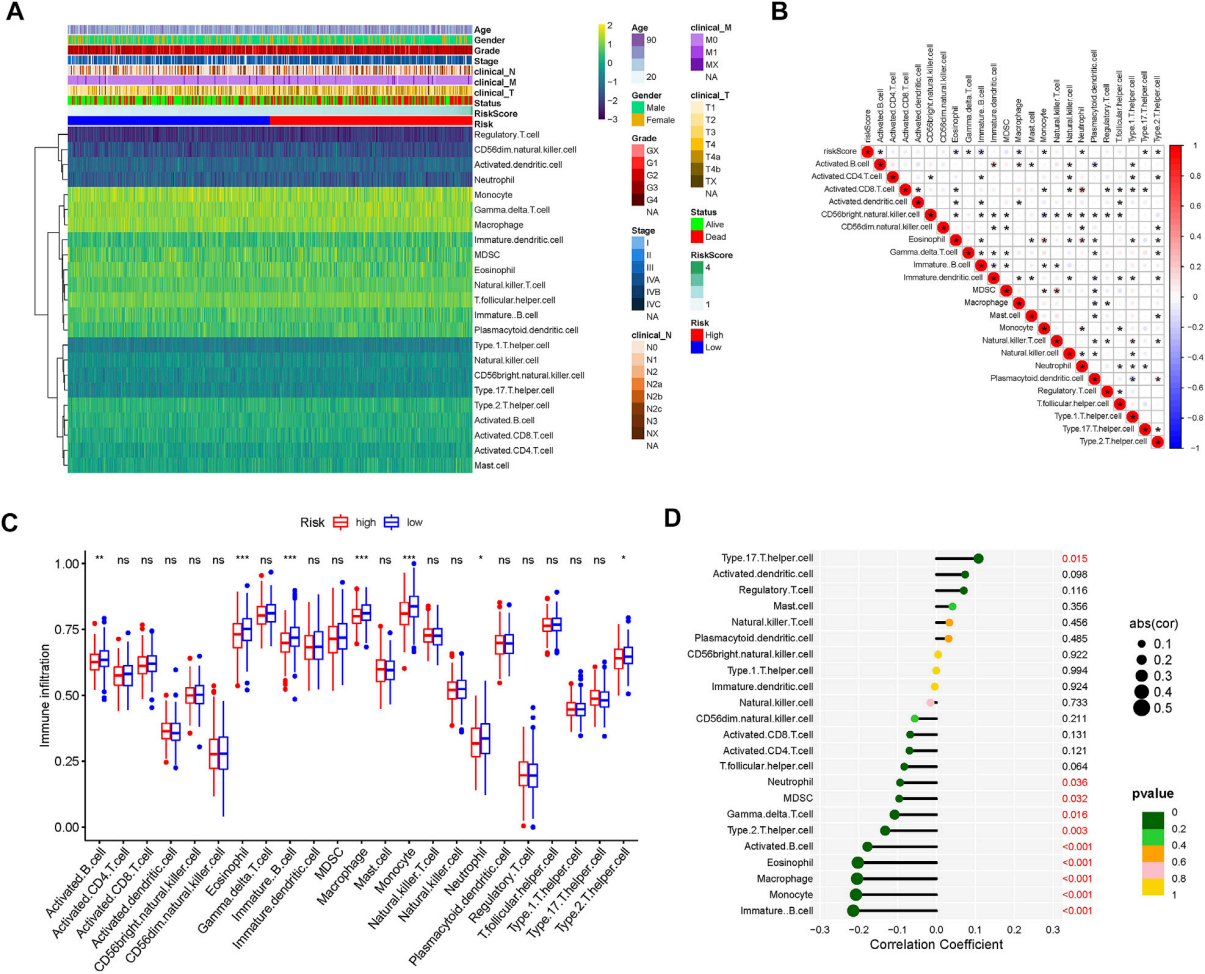
Tumor-infiltrating immune cell landscape estimation. **(A)** Heatmap of 23 infiltrating immune cell types in the TCGA-HNSC dataset. **(B)** Correlation between 23 infiltrating immune cell subtypes. Blue represents negative correlation and red represents positive correlation. *, *p* < 0.05. **(C)** Differences in the distribution of 23 tumor-infiltrating immune cells between the high- and low-risk groups. NS, not statistically significant; *p < 0.05, **p < 0.01, ***p < 0.001. **(D)** Correlation analysis of the risk score and 23 infiltrating immune cell subtypes.

To evaluate the landscape of the 23 infiltrating immune cell types in TCGA-HNSC, ssGSEA was used. Based on the clinical data of TCGA-HNSC and the risk score of each sample, a heatmap of the 23 infiltrating immune cell types were drawn (Figure 4A). Next, correlation analysis between tumor-infiltrating immune cells was performed. As shown in Figure 4B, the highest significantly positive correlation was between activated CD8 T cells and neutrophils, whereas the highest significantly negative correlation was between CD56bright natural killer cells and monocytes. The comparison analysis of tumor-infiltrating immune cell abundance between the different risk groups showed that the presence of activated and immature B cells, eosinophils, macrophages, monocytes, neutrophils, and Th2 cells were significantly reduced in the high-risk group (Figure 4C). Finally, we analyzed the correlation between 23 infiltrating immune cell types and the risk scores. The results showed that Th17 cells (*p* = 0.015) were the only significantly positively correlated cell type with the risk score, whereas the risk score had a significantly negative correlation with immature B cells, monocytes, macrophages, eosinophils, activated B cells, Th2 cells, gamma delta T cells, MDSCs, and neutrophils (Figure 4D). Collectively, our results show that the 7 cell types (activated B cells, eosinophils, immature B cells, macrophages, monocytes, neutrophils, and Th2 cells) may play an important role in the matrisome-related HNSCC microenvironment.

### 3.4 Somatic mutation profiles in the different risk groups

**FIGURE 5:**
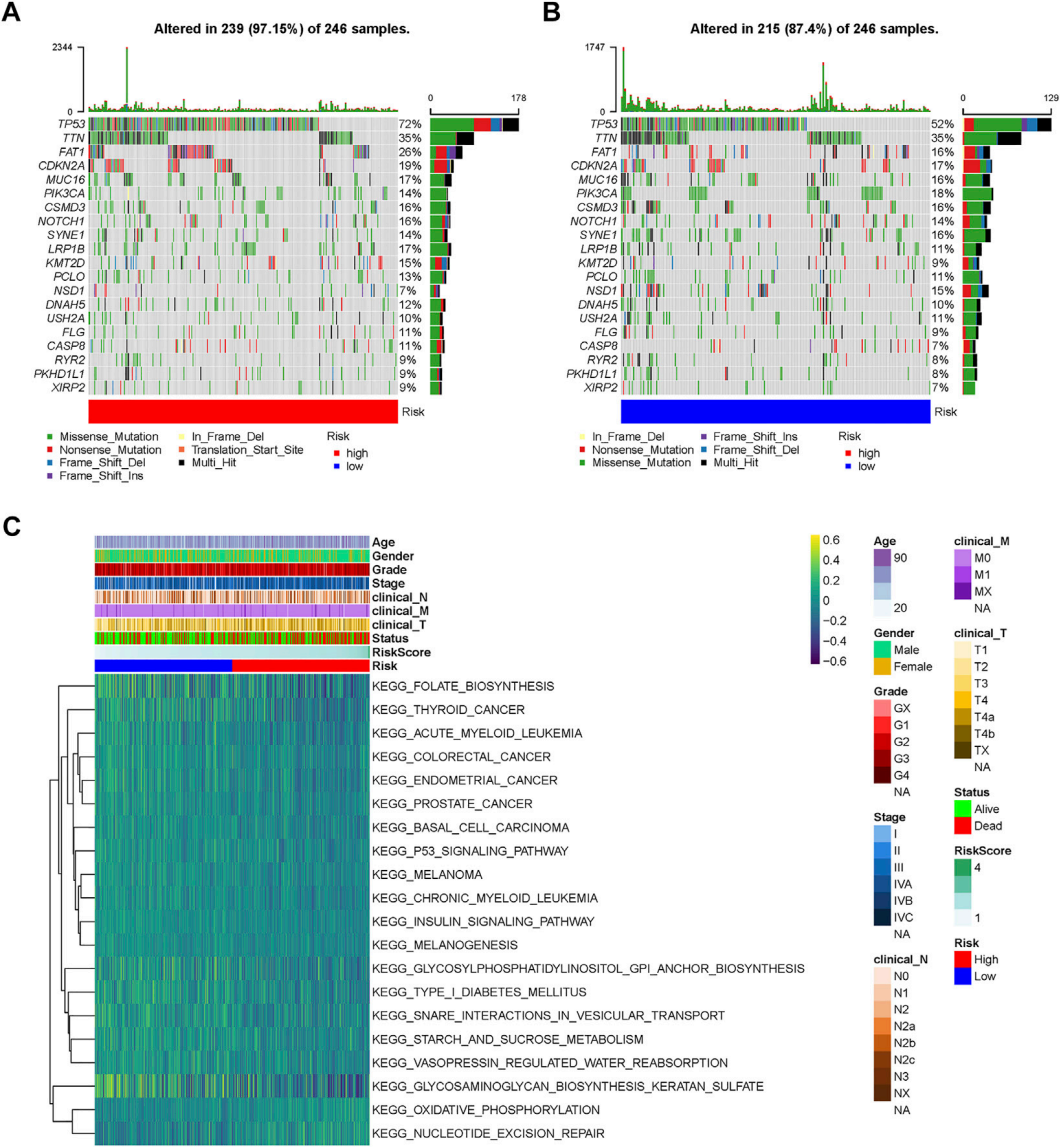
Landscape of mutation profiles and pathway enrichment between high and low-risk HNSCC patients. Waterfall plots represent mutation information in each sample of the **(A)** high- and **(B)** low-risk group. **(C)** Heatmap of KEGG analysis based on risk scores in the TCGA-HNSC dataset. KEGG, Kyoto Encyclopedia of Genes and Genomes; GSVA, Gene Set Variation Analysis.

Furthermore, the somatic mutation profiles of TCGA-HNSC patients were used to explore common somatic mutations in the high- and low-risk groups. Among these patients, 239 (97.15%) belonged to the high-risk group, and 215 (87.40%) belonged to the low-risk group. The frequency of gene mutations was generally higher in the high-risk group compared to the low-risk group. Alterations in the mutation landscape in high- or low-risk group were as follows: eight genes were mutated in >15% of tissues with high-risk score: *TP53* (72%), *TTN* (35%), *FAT1* (26%), *CDKN2A* (19%), *MUC16* (17%), *CSMD3* (16%), *NOTCH1* (16%), and *LRP1B* (17%), while eight genes were mutated in >15% of tissues with low-risk score: *TP53* (52%), *TTN* (35%), *FAT1* (16%), *CDKN2A* (17%), *MUC16* (16%), *PIK3CA* (18%), *CSMD3* (16%), and *SYNE1* (16%). Notably, *TP53* was one of the most commonly mutated genes in cancer, occurring more frequently in the high- (72%) than in the low-risk group (52%) (Figures 5A,B).

### 3.5 Functional enrichment analysis of prognostic genes

GSVA was performed to identify prognosis-related KEGG pathways. As shown in the heatmap, the high-risk group was significantly enriched in the P53 signaling pathway, thyroid cancer, colorectal cancer, endometrial cancer, prostate cancer, basal cell carcinoma, and melanoma KEGG pathways, while glycosaminoglycan biosynthesis keratan sulfate, glycosylphosphatidylinositol GPI anchor biosynthesis, and folate biosynthesis KEGG pathways were significantly enriched in the low-risk group (Figure 5C). Therefore, gene expression in the high-risk group was significantly enriched in tumor-related pathways.

### 3.6 Validation of protein expressions of prognostic genes

**FIGURE 6:**
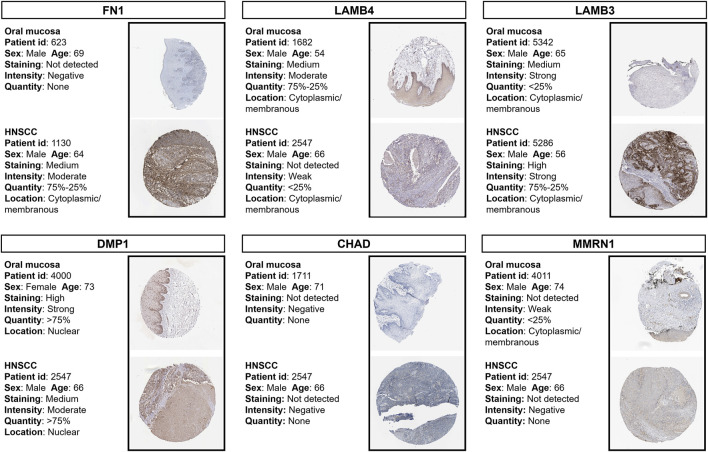
Immunohistochemistry staining of prognostic proteins based on the HPA. Protein expression levels of FN1, LAMB4, LAMB3, DMP1, CHAD and MMRN1 in tumor and normal tissues. HPA, Human Protein Atlas.

Using HPA online datasets, we then verified the protein expression of these prognostic genes through IHC. The FN1, LAMB3, and DMP1 proteins were found to be upregulated, while the LAMB4, CHAD, and MMRN1 proteins were found to be downregulated in HNSCC samples compared with the normal controls (Figure 6). These findings were consistent with our results obtained using the TCGA dataset.

### 3.7 Single-cell transcriptomic analysis of the prognostic genes

**FIGURE 7:**
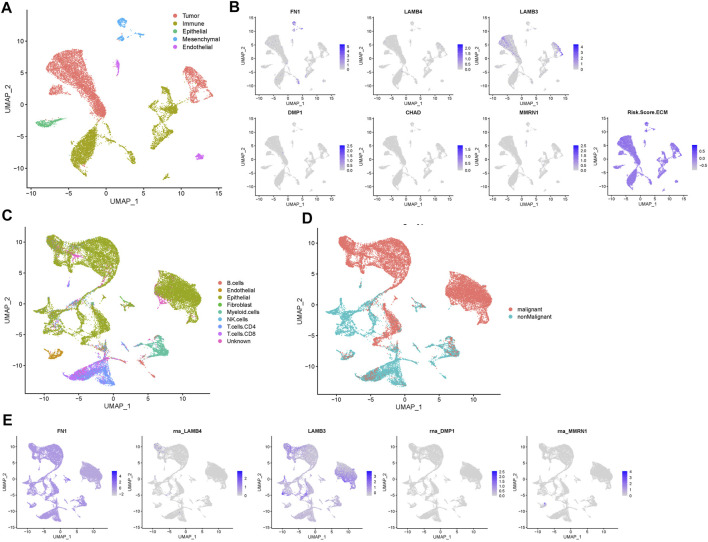
Prognostic expression profile based on single-cell sequencing analysis. **(A)** Composition and distribution of single cells in the GSE150321 dataset. **(B)** Distribution of the gene expression of *FN1*, *LAMB4*, *LAMB3*, *DMP1*, *CHAD*, *MMRN1* and the risk score in scRNA-Seq cluster of LSCC cells. **(C)** Composition and distribution of single cells in the GSE172577 dataset. **(D)** UMAP plots labeled nine different cell clusters. **(E)** The distribution of the expression of *FN1*, *LAMB4*, *LAMB3*, *DMP1*, and *MMRN1* in a scRNA-Seq cluster of OSCC cells. LSCC, laryngeal squamous cell carcinoma; OSCC, oral squamous cell carcinoma; scRNA-Seq, single-cell RNA-sequencing; UMAP, uniform manifold approximation and projection.

Next, we used single-cell RNA-Seq data from the GSE150321 and GSE172577 datasets to further verify the relationship between the prognostic model and genes in HNSCC. For the GSE150321 dataset, which comprised data from two LSCC samples, a total of 5 cell clusters (tumor, immune, epithelial, mesenchymal, and endothelial cells) were identified based on previous literature (Song et al., 2020) (Figure 7A). We then calculated the risk score for each cell and plotted it in a UMAP plot and violin plots (Figure 7B and Supplementary Figure S4). LSCC tumor cells had higher risk scores compared to the non-tumor cells.

For the GSE172577 dataset, four OSCC samples, GSM5258385, GSM5258386, GSM5258387, and GSM5258388, were combined. The cell clusters were annotated manually based on the “scCancer” R package (Figures 7C,D). Notably, the expression profile of most prognostic genes was similar to that in LSCC, except that in GSE172577 C*HAD* was expressed at lower levels (Figure 7E and Supplementary Figure S5). Collectively, these results further supported that this signature of matrisome-related prognostic genes influences HNSCC progression.

### 3.8 LAMB4 affects tumor cell proliferation and migration

**FIGURE 8:**
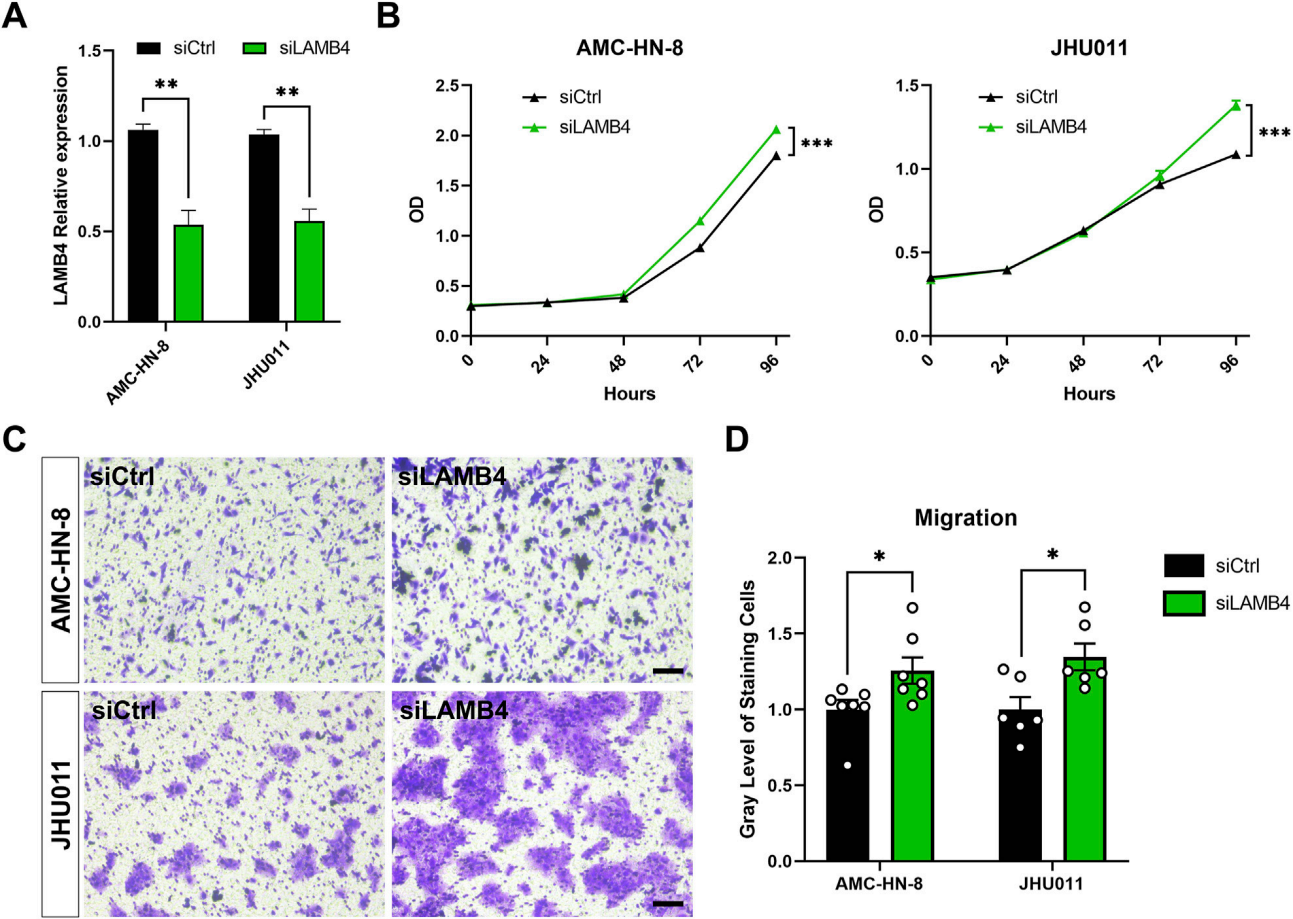
Silencing *LAMB4* in HNSCC cell lines promoted cell proliferation and migration. **(A)** AMC-HN-8 and JHU011 cells were transfected with siRNAs targeting *LAMB4* or control siRNAs (siCtrl) for 48 h. The expression of *LAMB4* was examined by RT-PCR. **(B)** AMC-HN-8 and JHU011 cells were transfected with siRNAs targeting *LAMB4* or control siRNAs (siCtrl), then cell proliferation was determined by CCK-8 assay. **(C)** The migration abilities of AMC-HN-8 and JHU011 cells after transfected with siRNAs targeting *LAMB4* or control siRNAs (siCtrl) were detected by Transwell assays. Bar, 100 μm. **(D)** Quantification of Transwell assay. *p < 0.05, **p < 0.01.

Based on the previous analysis results, we selected *LAMB4* for further analysis since it was one of the six prognostic genes that is less researched in the context of HNSCC. *LAMB4* siRNA was used to silence *LAMB4* expression in the AMC-HN-8 and JHU011 cell lines (Figure 8A). The CCK-8 assay demonstrated that silenced *LAMB4* levels promoted both AMC-HN-8 and JHU011 cell proliferation (Figure 8B). We also detected the migration change after *LAMB4* silencing. Transwell assays showed that *LAMB4* knockdown promoted migration in both LSCC and OSCC cell lines (Figures 8C,D). Therefore, as a potential tumor suppressor gene, silencing *LAMB4* promoted the proliferation and migration of HNSCC cell lines.

## 4 Discussion

In the current study, using the gene expression profile of TCGA-HNSC, we identified DEGs. Additionally, a list of core matrisome genes was obtained from the Matrisome Project. After intersecting the DEGs in TCGA-HNSC and common core matrisome genes, 52 DEGs were identified as differentially expressed matrisome-related genes. Subsequent univariate and multivariate Cox regression and LASSO analyses were performed to establish a prognostic model consisting of a risk signature comprising six genes (*FN1*, *LAMB4*, *LAMB3*, *DMP1*, *CHAD*, and *MMRN1*). This model was validated using two independent datasets for LSCC and OSCC. Among these six prognostic genes, five (*FN1*, *LAMB4*, *LAMB3*, *DMP1*, and *MMRN1*) were classified as ECM glycoprotein-coding genes, and one (*CHAD*) was classified as a proteoglycan-coding gene. Tumor-infiltrating immune cell landscape, somatic mutation, GSVA, and immunohistochemistry analyses based on the prognostic model and genes were performed. Single-cell transcriptomic analysis was used to verify the expression patterns of these prognostic genes and models.

Tumor ECM remodeling increases cancer cell proliferation and survival. We have previously shown that most of the core ECM genes were highly expressed in LSCC tissues and were enriched in the DEGs of the GSE142083 dataset (Huang et al., 2021). Here, we employed the TCGA-HNSC dataset and confirmed that both subgroups of HNSCC showed ECM expression enrichment. Therefore, therapies targeting ECM remodeling may be clinically promising. However, ECM remodeling involves two important modifications: stiffness and degradation. Abnormal ECM stiffness is important for epithelial-mesenchymal transition (Alonso-Nocelo et al., 2018), immune cell differentiation (Lu et al., 2012), and the promotion of tumor cell proliferation (Chen et al., 2018). In contrast, to help tumor cells break collagen migration barriers, ECM degradation is involved in tumor migration and invasion and angiogenesis induction (Winkler et al., 2020). Therefore, these two processes are interrelated, and therapies targeting either or both processes without inducing the other one would be challenging.

*FN1* encodes fibronectin, an ECM glycoprotein involved in cell adhesion and migration. FN1 plays a critical role in the development of ovarian (Liang et al., 2020), thyroid (Geng et al., 2021), renal (Waalkes et al., 2010), cervical (Zhou et al., 2019a), breast (Huang et al., 2022), and gastric cancers (Han et al., 2020). Liu et al. (2020) reported that the expression level of *FN1* is also correlated with a poor HNSCC prognosis and that p62/SQSTM1 may participate as an autophagy adapter in the autophagy-lysosome pathway for FN1 degradation. Mechanistically, fibronectin, which is mainly secreted by cancer-associated fibroblasts (CAFs), is reorganized by CAFs through increased contractility and traction forces. This reorganization ultimately promotes CAF–cancer cell interactions and leads to directional cancer cell migration (Erdogan et al., 2017). Hence, a comprehensive understanding of the functions of fibronectin in the TME is crucial.

Laminin is a multidomain glycoprotein composed of α, β, and γ subunits. In mammals, four genes (*LAMB1*—*4*) encode four different β-chains of laminin (Yurchenco et al., 2018). Among these, *LAMB3* has been shown to play a role in cancer development, and the involvement of laminin-332 (assembled by three subunits, α3, β3, and γ2) in cancer pathogenesis has been extensively reported. Hagedorn et al. observed that laminin-332 was highly expressed in different LSCC stages and was distributed within tumor cells at the tumor invasion front (Hagedorn et al., 2001). Furthermore, high levels of laminin-332 are associated with tumor invasion (Nordemar et al., 2001). In contrast to *LAMB3*, the role of *LAMB4* in cancer has not been intensively investigated. Choi et al. demonstrated that loss of expression of *LAMB4*, a candidate tumor suppressor gene, was identified in 17%–32% of gastric and colorectal cancers (Choi et al., 2015). Here, we verified for the first time that knocking down *LAMB4* promotes proliferation and migration in HNSCC cell lines (Figures 8B–D). Consistent with these previous studies, our analysis of *LAMB3* and *LAMB4* from the TCGA-HNSC dataset indicated that both *LAMB3* and *LAMB4* could serve as prognostic markers for squamous cell carcinoma. However, the underlying mechanism of these two genes, especially *LAMB4*, requires further investigation.

DMP1, also called dentin matrix protein 1, is an ECM protein that belongs to the small integrin-binding ligand N-linked glycoprotein (SIBLING) family (Fisher et al., 2001). DMP1 is mainly expressed in bones and dentin and in non-mineralized tissues, such as the brain, kidney, and salivary glands. Under normal conditions, DMP1 controls the maturation of odontoblasts and osteoblasts by functioning as a transcriptional co-factor (Narayanan et al., 2003). However, DMP1 was found to be significantly elevated in different cancer types (Fisher et al., 2004). Karadag et al. (2005) suggested that DMP1 could enhance the invasion potential of cancer cells by bridging MMP-9 to the colon cell surface through α_v_β_3_-integrin, α_v_β_5_-integrin, and/or CD44. Our results suggest that *DMP1* may also serve as a prognosis-associated gene in OSCC and LSCC. Further studies are required to determine the role of *DMP1* in HNSCC progression.

*CHAD* encodes the cartilage matrix protein chondroadherin, which promotes cell attachment by binding to α_2_β_1_-integrin and syndecans. Additionally, CHAD plays an important role in the ECM of mineralized tissues. In patients with osteoporosis and ovariectomized mice, CHAD was downregulated, thus inhibiting preosteoclast motility and bone resorption (Capulli et al., 2014). However, few studies have reported the relationship between *CHAD* expression and cancer development. Deng et al. (2017) showed that low *CHAD* expression was significantly associated with poor survival in hepatocellular carcinoma. In our analysis, *CHAD* was significantly downregulated in HNSCC samples, and its low expression was associated with a high-risk score and poor prognosis in both patients with LSCC and OSCC (Figure 4). Nevertheless, future studies are warranted to clarify the mechanisms underlying chondroadhesion in HNSCC.

*MMRN1* encodes multimerin 1, a member of the elastin microfibrillar interface protein family. Under *in vivo* conditions, multimerin one is expressed in platelets and the endothelium and may be involved in cellular adhesion via integrin receptors (Colombatti et al., 2011). As a platelet protein, multimerin 1 has shown good predictive value as a biomarker for acute myeloid leukemia (Laszlo et al., 2015). Moreover, *Mmrn1*-defected mice showed significantly impaired platelet adhesion and thrombus formation in a ferric chloride injury model compared to the wild-type (Leatherdale et al., 2021). *MMRN1* is implicated in several types of cancers, including non-small cell lung cancer (Valk et al., 2010), thyroid carcinoma (Wang et al., 2018; Yang et al., 2021; Zhang et al., 2019), ovarian cancer (Huang et al., 2012), and cervical cancer (Chokchaichamnankit et al., 2019). In this study, we identified *MMRN1* as a hub gene for HNSCC, and its low expression was associated with a poor prognosis. However, its role in HNSCC remains unclear.

This study had some limitations. First, there were only 44 adjacent normal samples versus 502 tumor samples in TCGA-HNSC, which may have led to a potential statistical error in DEGs screening. Second, the validation cohorts from the GEO database lacked sufficient clinical data; thus, related validation analysis of TNM stage in our prognostic model could not be performed. Third, further experiments are required to explore the mechanism of these prognostic genes in HNSCC and the effectiveness of this prognostic model in clinical practice. Fourth, human papillomavirus (HPV) infection has been proved playing an important role in HNSCC induction (Marur et al., 2010). Our model showed good prediction in HPV negative HNSCC patients (Supplementary Figures S6A–D). However, due to the deficiency of HPV positive samples in TCGA-HNSC dataset, we could not perform an effective prognostic analysis in this cohort (Supplementary Figures S6E–H). More HPV positive samples are required to validate our prognostic model.

In summary, we revealed that matrisome gene expression are associated with HNSCC survival, and established a novel risk score prognostic model based on a signature of six differentially expressed matrisome-related genes (*FN1*, *LAMB4*, *LAMB3*, *DMP1*, *CHAD*, and *MMRN1*), which may also act as potential therapeutic targets for HNSCC.

## Data Availability

The datasets presented in this study can be found in online repositories. The names of the repository/repositories and accession numbers can be found in the article/Supplementary Material.

## Abbreviations

AUC, area under the curve; BCV, biological coefficient of variation; BP, biological processes; CAFs, cancer-associated fibroblasts; DCs, dendritic cells; DEGs, differentially expressed genes; DMEM, dulbecco’s modified eagle’s medium; ECM, extracellular matrix; FBS, fetal bovine serum; FC, fold change; GEO, gene expression omnibus; GO, gene ontology; HNSCC, head and neck squamous cell carcinoma; HPA, the human protein atlas; IHC, immunohistochemistry; KEGG, kyoto encyclopedia of genes and genomes; LASSO, least absolute shrinkage and selection operator; LSCC, laryngeal squamous cell carcinoma; MDSCs, myeloid-derived suppressor cells; OS, overall survival; OSCC, oral squamous cell carcinoma; ROC, receiver operating characteristic; scRNA-Seq, single-cell RNA-sequencing; SIBLING, small integrin-binding ligand N-linked glycoprotein; ssGSEA, single-sample gene set enrichment analysis; TCGA-HNSC, the cancer genome atlas-head and neck squamous cell carcinoma; Th1, type 1 T helper; TME, tumor microenvironment; UCSC, the University of California, Santa Cruz; UMAP, uniform manifold approximation and projection.
