# Supplementary material for: Novel prognostic matrisome-related gene signature of head and neck squamous cell carcinoma
Source: Front Cell Dev Biol. 2022 Aug 23;10:884590. doi: 10.3389/fcell.2022.884590 (PMC9445128; doi:10.3389/fcell.2022.884590)
Supplement: Supplementary file 1 [file Table1.DOCX]

Supplementary Material

# Supplementary Figures and Tables

## Supplementary Figures


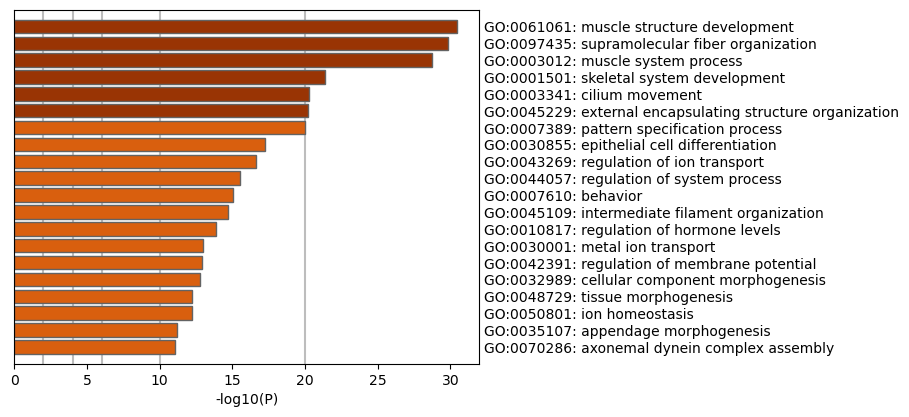


**Supplementary Figure S1.** GO BP analysis of the DEGs in TCGA-HNSC. GO, Gene ontology; BP, biological processes; DEGs, Differentially expressed genes.


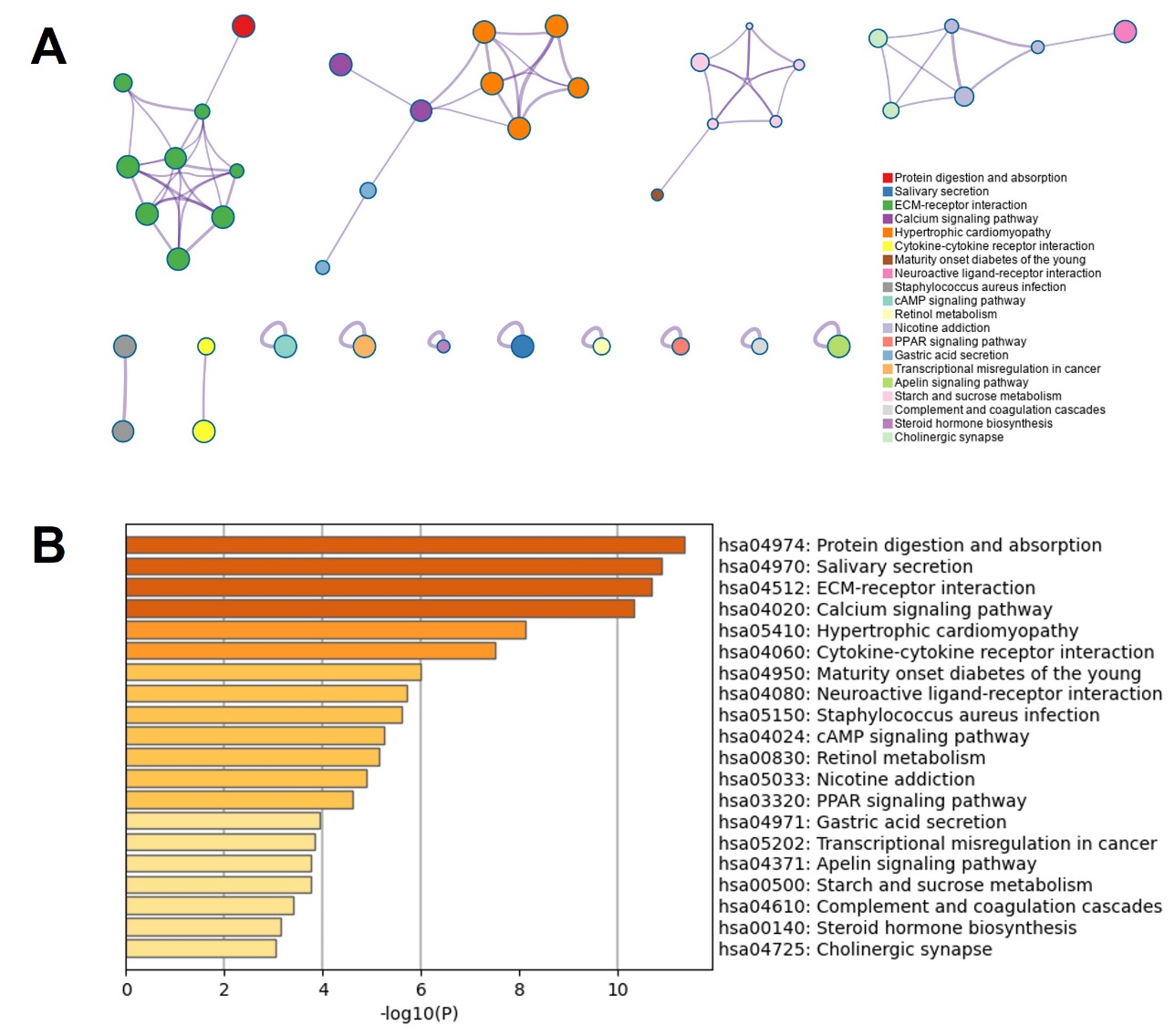


**Supplementary Figure S2.** KEGG analysis of the DEGs in TCGA-HNSC ranked by **(A)** cluster or **(B)** *p*-value. KEGG, Kyoto Encyclopedia of Genes and Genomes; DEGs, Differentially expressed genes.


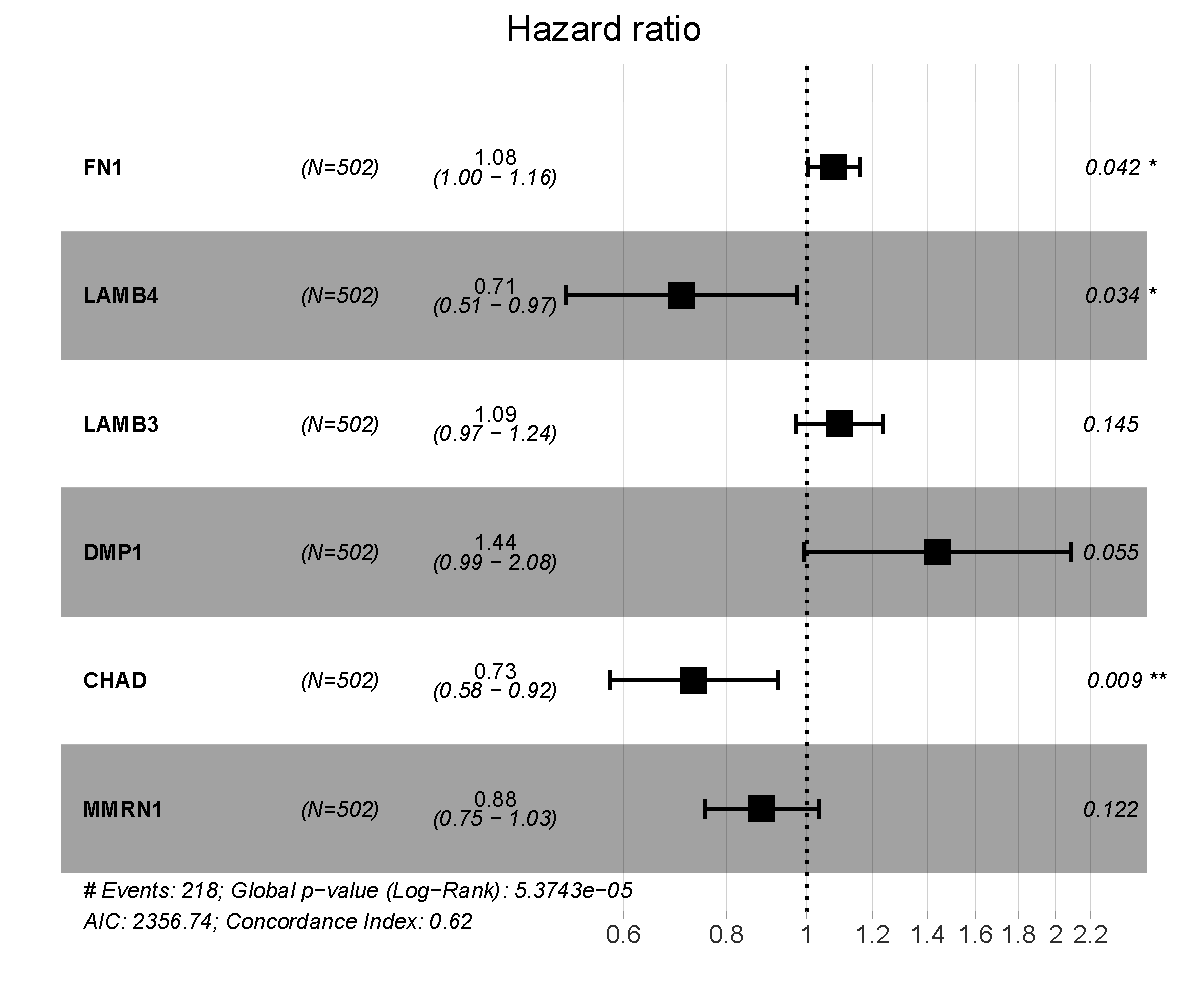


**Supplementary Figure S3.** The multivariate analysis of risk factors in training cohort.


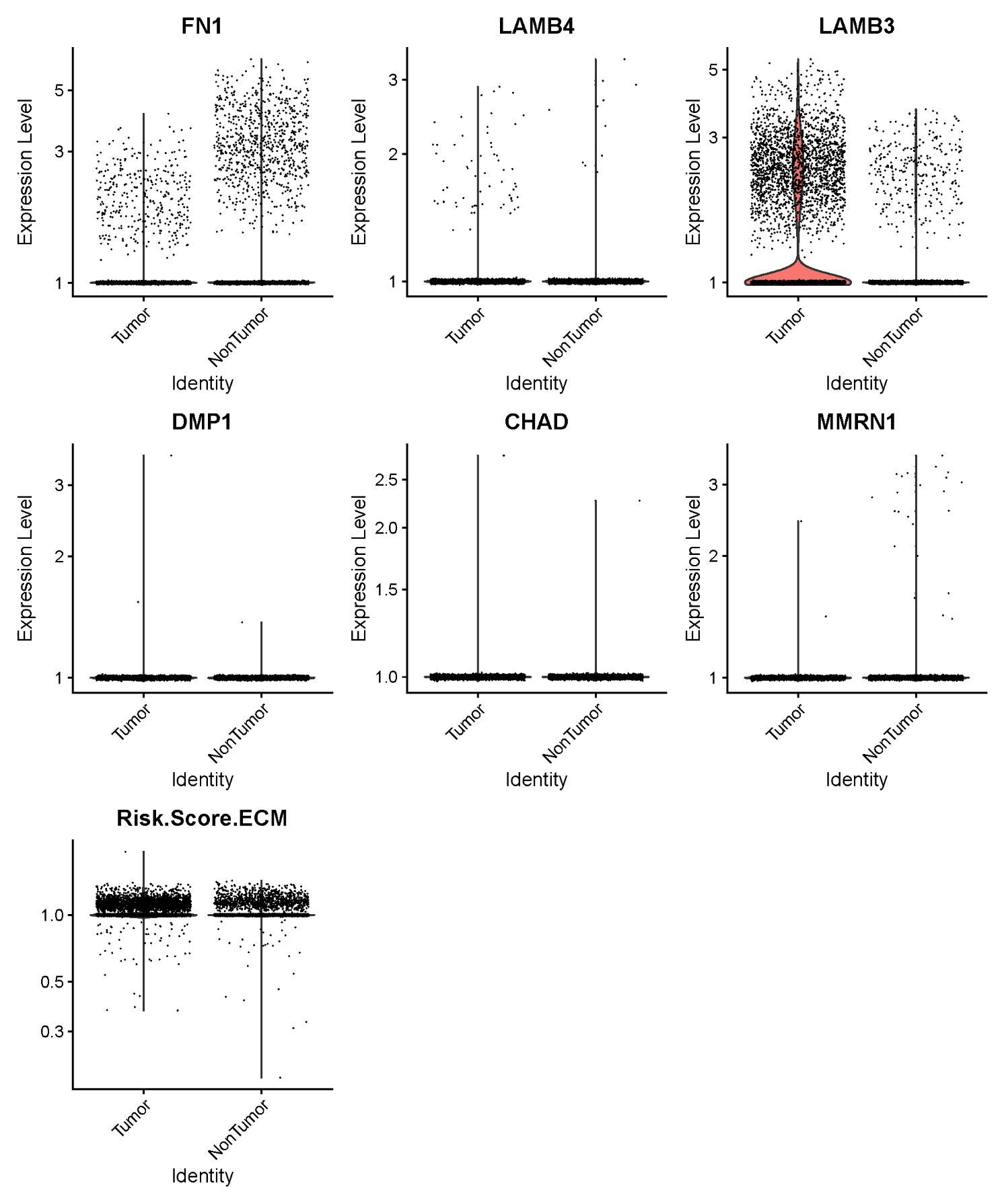


**Supplementary Figure S4.** The violin plots of the expression profile of *FN1*, *LAMB4*, *LAMB3*, *DMP1*, *CHAD*, *MMRN1* and risk score in GSE150321.


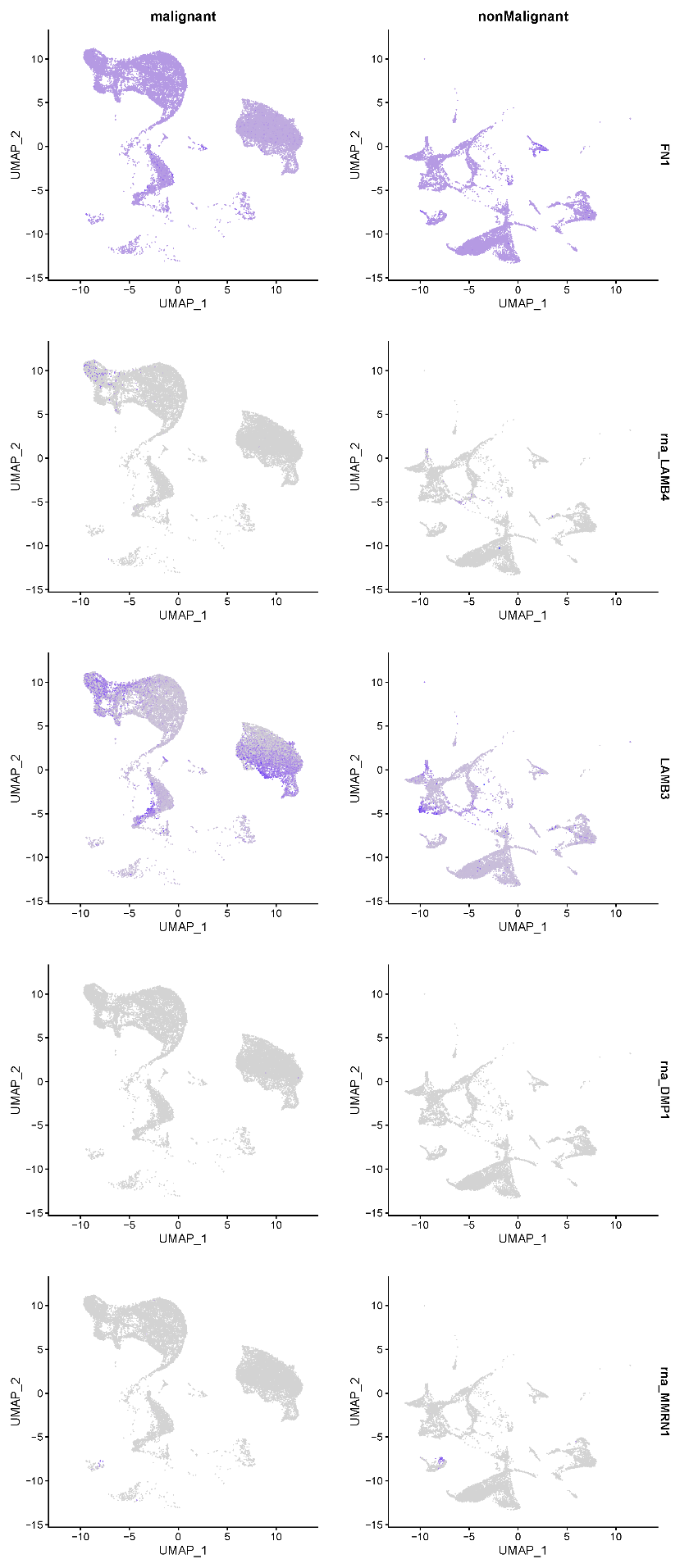


**Supplementary Figure S5.** The expression profile of *FN1*, *LAMB4*, *LAMB3*, *DMP1* and *MMRN1* in GSE172577.


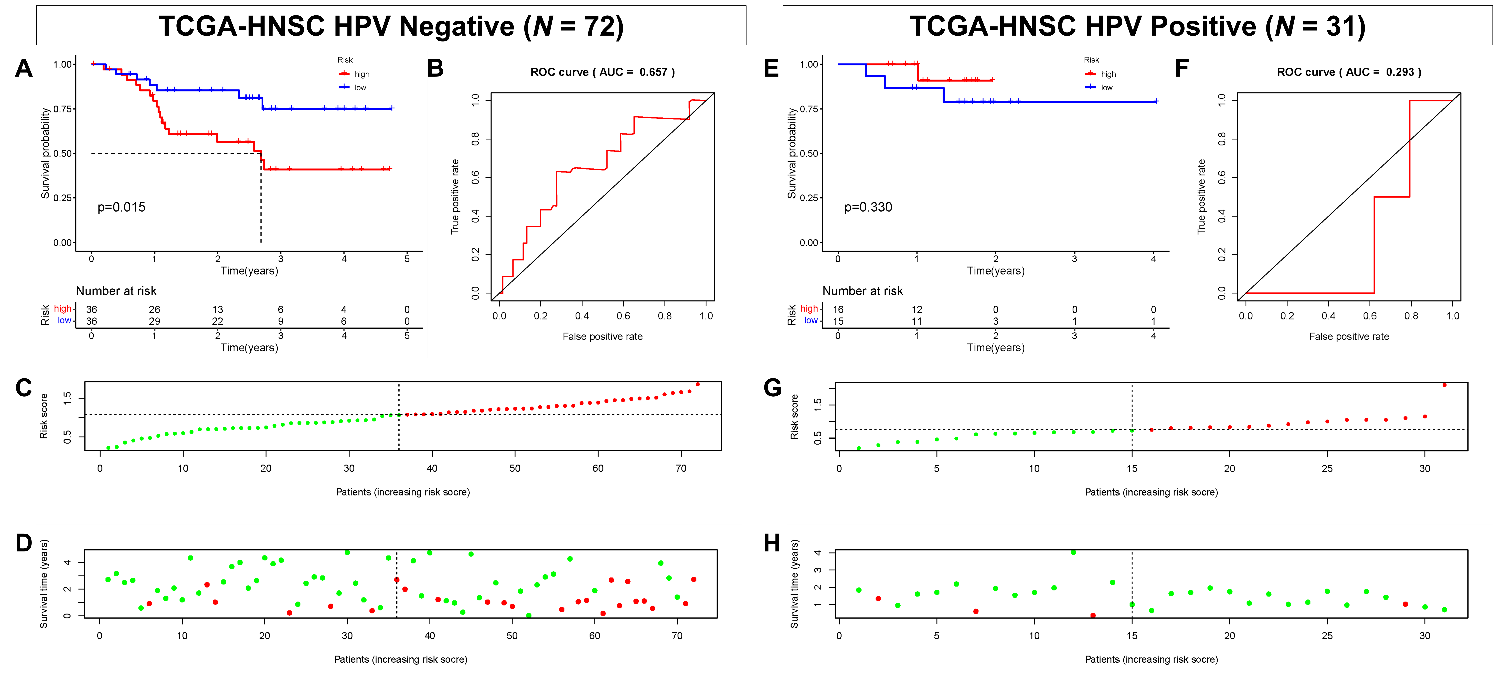


**Supplementary Figure S6.** Prognostic analysis in (**A-D**) HPV negative or (**E-H**) positive cohorts of TCGA-HNSC. HPV, human papillomavirus.

## Supplementary Tables

**Supplementary Table S1. Basic characteristics of the study datasets**

|  | **TCGA-HNSC** | **GSE27020** | **GSE42743** |
| --- | --- | --- | --- |
| **No. of Normal** | 44 | 0 | 29 |
| **No. of Tumor** | 502 | 109 | 74 |
| **Sample Type** | Primary solid tumors | Primary solid tumors | Primary solid tumors |
| **Age at diagnosis (%)** |  |  |  |
| **≤65** | 325 (64.74) | 61 (55.96) | 53 (71.62) |
| **>65** | 176 (35.06) | 48 (44.04) | 21 (28.38) |
| **Gender (%)** |  |  |  |
| **Female** | 134 (26.69) | NA | 16 (21.62) |
| **Male** | 368 (73.31) | NA | 58 (78.38) |
| **Grade (%)** |  |  |  |
| **G1** | 62 (12.35) | 42 (38.53) | NA |
| **G2** | 300 (59.76) | 49 (44.95) | NA |
| **G3** | 119 (23.71) | 16 (14.68) | NA |
| **G4** | 2 (0.40) | NA | NA |
| **GX** | 16 (3.19) | NA | NA |
| **Stage (%)** |  |  |  |
| **I** | 19 (3.78) | NA | NA |
| **II** | 95 (18.92) | NA | NA |
| **III** | 102 (20.32) | NA | NA |
| **IV** | 272 (54.18) | NA | NA |
| **Survival status** |  |  |  |
| **Survival day (median)** | 637 | 1050 | 441.5 |
| **Ending (%)** |  |  |  |
| **Death** | 218 (43.43) | 34 (31.19) | 42 (56.76) |
| **Survival** | 284 (56.57) | 75 (68.81) | 32 (43.24) |

**Supplementary Table S2. List of 190 common core matrisome genes**

| *ABI3BP* | *ACAN* | *ADIPOQ* | *AEBP1* | *AGRN* |
| --- | --- | --- | --- | --- |
| *AMBN* | *AMELX* | *AMELY* | *ASPN* | *BCAN* |
| *BGN* | *CHAD* | *CILP* | *COCH* | *COL10A1* |
| *COL11A1* | *COL11A2* | *COL13A1* | *COL14A1* | *COL15A1* |
| *COL16A1* | *COL17A1* | *COL18A1* | *COL19A1* | *COL1A1* |
| *COL1A2* | *COL21A1* | *COL2A1* | *COL3A1* | *COL4A1* |
| *COL4A2* | *COL4A3* | *COL4A4* | *COL4A5* | *COL4A6* |
| *COL5A1* | *COL5A2* | *COL5A3* | *COL6A1* | *COL6A2* |
| *COL6A3* | *COL7A1* | *COL8A1* | *COL8A2* | *COL9A1* |
| *COL9A2* | *COL9A3* | *COLQ* | *COMP* | *CRELD1* |
| *CRELD2* | *CRISPLD2* | *DCN* | *DMBT1* | *DMP1* |
| *DPT* | *DSPP* | *ECM1* | *ECM2* | *EDIL3* |
| *EFEMP1* | *EFEMP2* | *ELN* | *ELSPBP1* | *EMID1* |
| *EMILIN1* | *EMILIN2* | *EPYC* | *ESM1* | *FBLN1* |
| *FBLN2* | *FBLN5* | *FBN1* | *FBN2* | *FGA* |
| *FGB* | *FGG* | *FGL1* | *FGL2* | *FMOD* |
| *FN1* | *FNDC8* | *FRAS1* | *GAS6* | *HAPLN1* |
| *HAPLN2* | *HSPG2* | *IBSP* | *IGFALS* | *IGFBP1* |
| *IGFBP2* | *IGFBP3* | *IGFBP4* | *IGFBP5* | *IGFBP6* |
| *IGFBP7* | *IMPG1* | *IMPG2* | *KERA* | *LAMA1* |
| *LAMA2* | *LAMA3* | *LAMA4* | *LAMA5* | *LAMB1* |
| *LAMB2* | *LAMB3* | *LAMB4* | *LAMC1* | *LAMC2* |
| *LAMC3* | *LGI1* | *LGI2* | *LTBP1* | *LTBP2* |
| *LTBP3* | *LTBP4* | *LUM* | *MATN1* | *MATN3* |
| *MATN4* | *MEPE* | *MFAP1* | *MFAP2* | *MFAP3* |
| *MFAP4* | *MFAP5* | *MFGE8* | *MGP* | *MMRN1* |
| *MMRN2* | *MXRA5* | *NCAN* | *NDNF* | *NELL1* |
| *NELL2* | *NID1* | *NID2* | *NTN1* | *NTN3* |
| *NTNG1* | *NYX* | *OGN* | *OMD* | *PCOLCE* |
| *PCOLCE2* | *PODNL1* | *POMZP3* | *POSTN* | *PRELP* |
| *PRG2* | *PRG3* | *PRG4* | *PXDN* | *RELN* |
| *SBSPON* | *SLIT1* | *SLIT2* | *SLIT3* | *SNED1* |
| *SPARC* | *SPARCL1* | *SPOCK1* | *SPOCK2* | *SPOCK3* |
| *SPON1* | *SPP1* | *SRGN* | *SRPX* | *SRPX2* |
| *SVEP1* | *TECTA* | *TGFBI* | *THBS1* | *THBS2* |
| *THBS3* | *THBS4* | *THSD4* | *TINAGL1* | *TNC* |
| *TNFAIP6* | *TNN* | *TNR* | *TSKU* | *VCAN* |
| *VWA1* | *VWA5A* | *VWA7* | *VWF* | *ZP2* |

**Supplementary Table S3. Univariate Cox regression analysis in the train cohort**

| **Characteristics** | **HR** | **95% CI** | ***p*-value** |
| --- | --- | --- | --- |
| *MATN3* | 1.096 | 0.984-1.220 | 0.095† |
| *LAMA1* | 1.069 | 0.968-1.179 | 0.185 |
| *NCAN* | 0.833 | 0.435-1.592 | 0.581 |
| *PRG4* | 1.054 | 0.929-1.194 | 0.410 |
| *PXDN* | 1.057 | 0.965-1.156 | 0.233 |
| *LAMC2* | 1.088 | 1.002-1.179 | 0.043† |
| *HAPLN1* | 0.876 | 0.720-1.064 | 0.183 |
| *NID1* | 1.073 | 0.979-1.174 | 0.129 |
| *COL6A3* | 0.986 | 0.912-1.066 | 0.725 |
| *FN1* | 1.067 | 1.000-1.137 | 0.049† |
| *SPP1* | 1.062 | 1.005-1.121 | 0.031† |
| *LAMB4* | 0.703 | 0.516-0.954 | 0.024† |
| *LAMB3* | 1.128 | 1.007-1.263 | 0.037† |
| *COL4A5* | 1.039 | 0.926-1.164 | 0.511 |
| *COL6A1* | 0.982 | 0.896-1.075 | 0.692 |
| *COL11A1* | 1.049 | 0.987-1.113 | 0.122 |
| *COL5A2* | 1.029 | 0.949-1.114 | 0.486 |
| *COL3A1* | 1.012 | 0.944-1.084 | 0.730 |
| *SPARC* | 1.022 | 0.933-1.119 | 0.634 |
| *COL4A1* | 1.016 | 0.909-1.133 | 0.784 |
| *DMP1* | 1.592 | 1.135-2.233 | 0.007† |
| *LUM* | 0.979 | 0.900-1.064 | 0.623 |
| *COL5A3* | 0.979 | 0.882-1.085 | 0.682 |
| *LAMA3* | 1.071 | 0.983-1.166 | 0.115 |
| *IBSP* | 1.105 | 1.001-1.219 | 0.048† |
| *CILP* | 1.024 | 0.922-1.135 | 0.659 |
| *FBN2* | 1.033 | 0.955-1.116 | 0.414 |
| *TNC* | 1.017 | 0.944-1.094 | 0.659 |
| *ADIPOQ* | 1.031 | 0.819-1.296 | 0.797 |
| *COL1A2* | 1.005 | 0.934-1.080 | 0.900 |
| *COL5A1* | 1.040 | 0.958-1.129 | 0.343 |
| *COL4A6* | 1.059 | 0.977-1.148 | 0.162 |
| *COL10A1* | 1.042 | 0.977-1.111 | 0.204 |
| *MFAP2* | 1.096 | 0.978-1.226 | 0.113 |
| *MGP* | 1.016 | 0.934-1.105 | 0.704 |
| *DMBT1* | 0.915 | 0.842-0.992 | 0.033† |
| *NID2* | 0.999 | 0.906-1.101 | 0.983 |
| *TGFBI* | 1.101 | 1.014-1.193 | 0.021† |
| *COL2A1* | 1.011 | 0.875-1.168 | 0.878 |
| *COL1A1* | 1.017 | 0.946-1.094 | 0.641 |
| *EPYC* | 0.982 | 0.876-1.100 | 0.754 |
| *COL7A1* | 0.936 | 0.823-1.064 | 0.316 |
| *COL8A1* | 0.990 | 0.908-1.078 | 0.820 |
| *COL4A2* | 1.004 | 0.889-1.132 | 0.950 |
| *ASPN* | 0.969 | 0.902-1.041 | 0.395 |
| *CHAD* | 0.745 | 0.597-0.928 | 0.009† |
| *OGN* | 0.986 | 0.886-1.095 | 0.788 |
| *POSTN* | 1.052 | 0.985-1.122 | 0.127 |
| *LGI1* | 1.286 | 0.239-6.908 | 0.769 |
| *MMRN1* | 0.868 | 0.750-1.003 | 0.055† |
| *COL13A1* | 1.048 | 0.873-1.257 | 0.617 |
| *NELL2* | 1.029 | 0.959-1.104 | 0.418 |

HR, hazard ratio; CI, confidence interval; †*p* < 0.10.
